# Supplementary figures and images for: LDLR c.415G > A causes familial hypercholesterolemia by weakening LDLR binding to LDL
Source: Lipids Health Dis. 2024 Mar 21;23:85. doi: 10.1186/s12944-024-02068-2 (PMC10956282; doi:10.1186/s12944-024-02068-2)

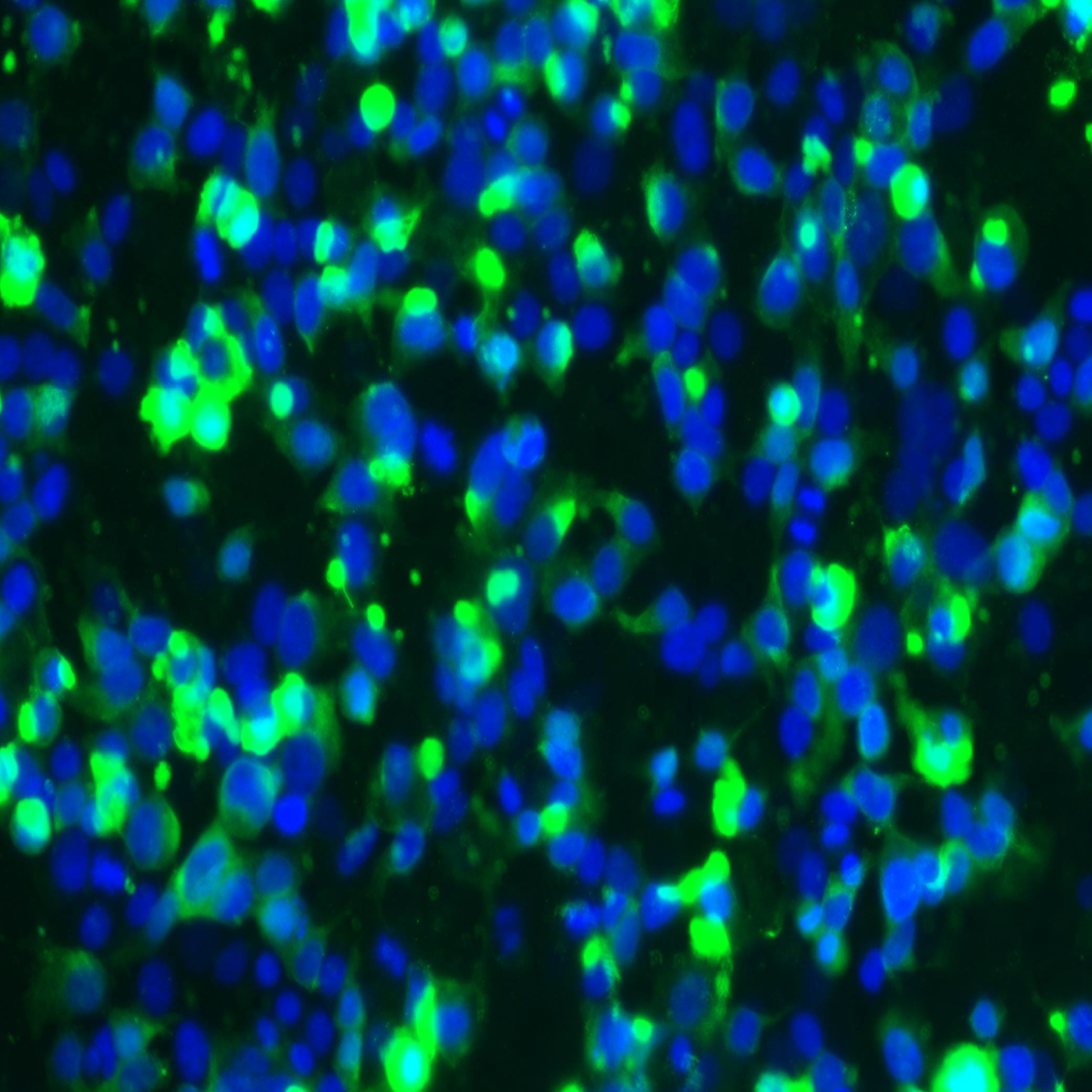

Supplement: Supplementary file 1 — Supplementary Material 1: The success rate of transfection. Double immunofluorescence staining of LDLR (green)and DAPI (blue) [file 12944_2024_2068_MOESM1_ESM.jpg]

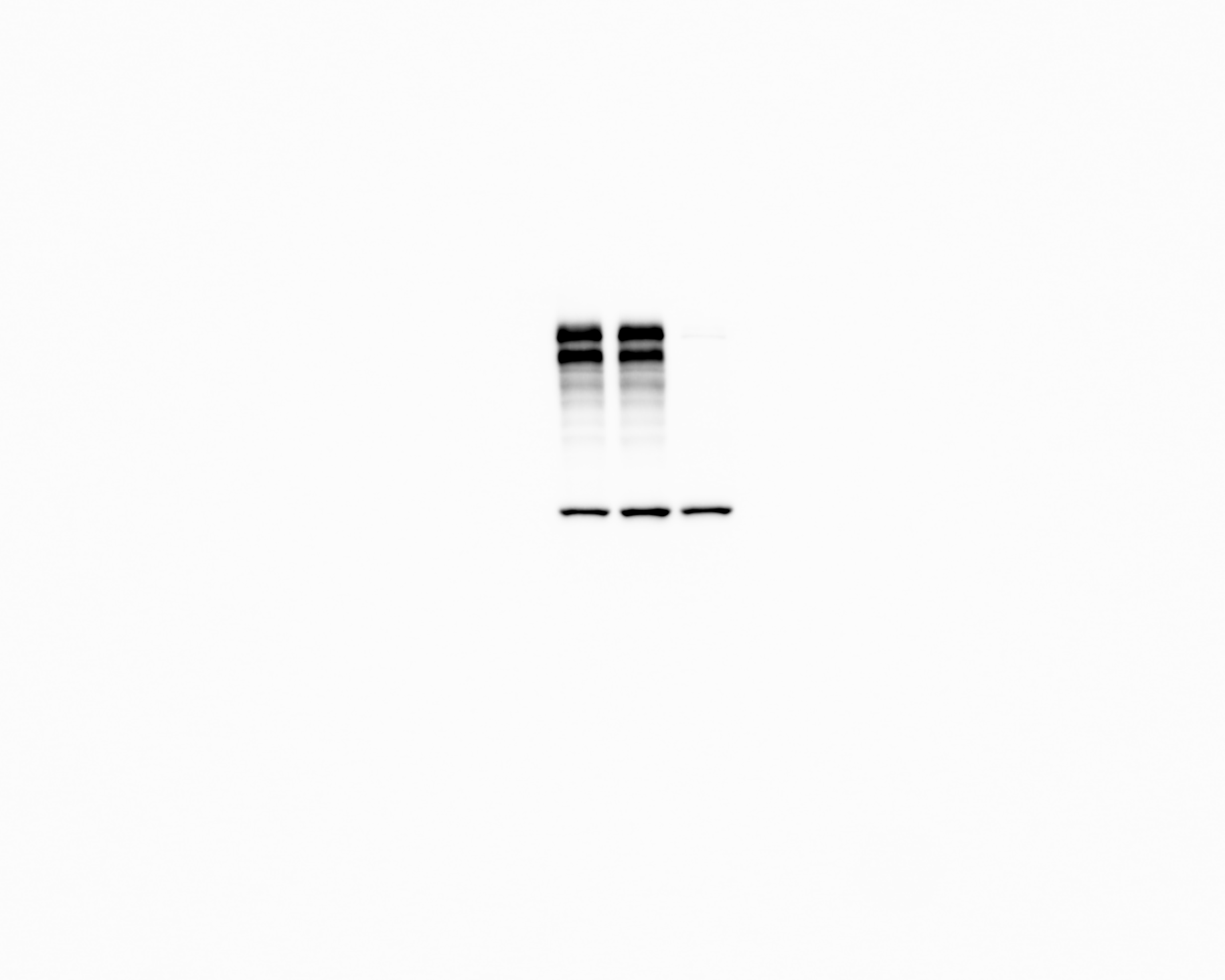

Supplement: Supplementary file 5 — Supplementary Material 5: The image of LDLR and Actin obtained through chemiluminescence [file 12944_2024_2068_MOESM5_ESM.tif]

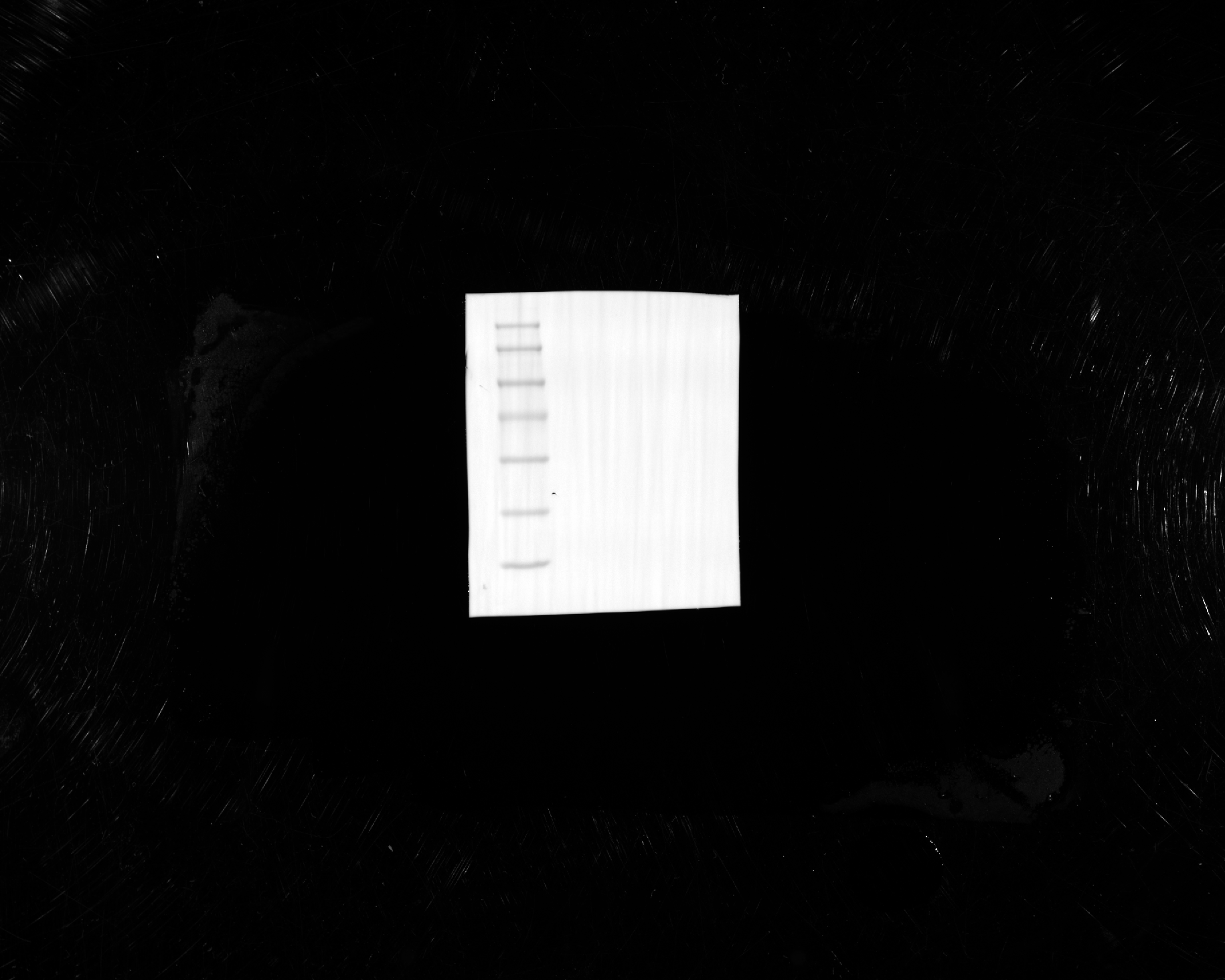

Supplement: Supplementary file 6 — Supplementary Material 6: The image of Marker obtained through colorimetric [file 12944_2024_2068_MOESM6_ESM.tif]

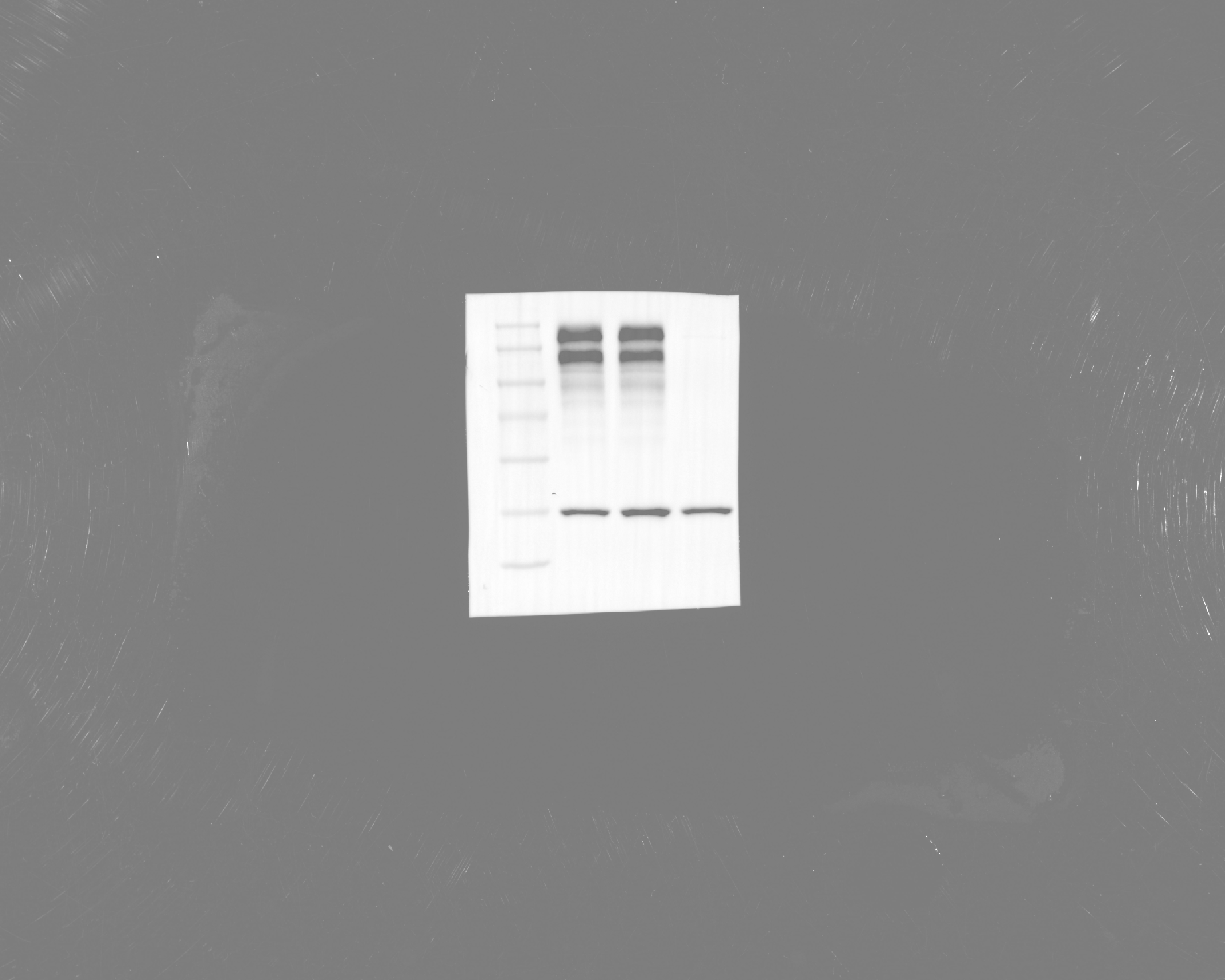

Supplement: Supplementary file 7 — Supplementary Material 7: the overlay image of LDLR, Actin and Marker [file 12944_2024_2068_MOESM7_ESM.tif]

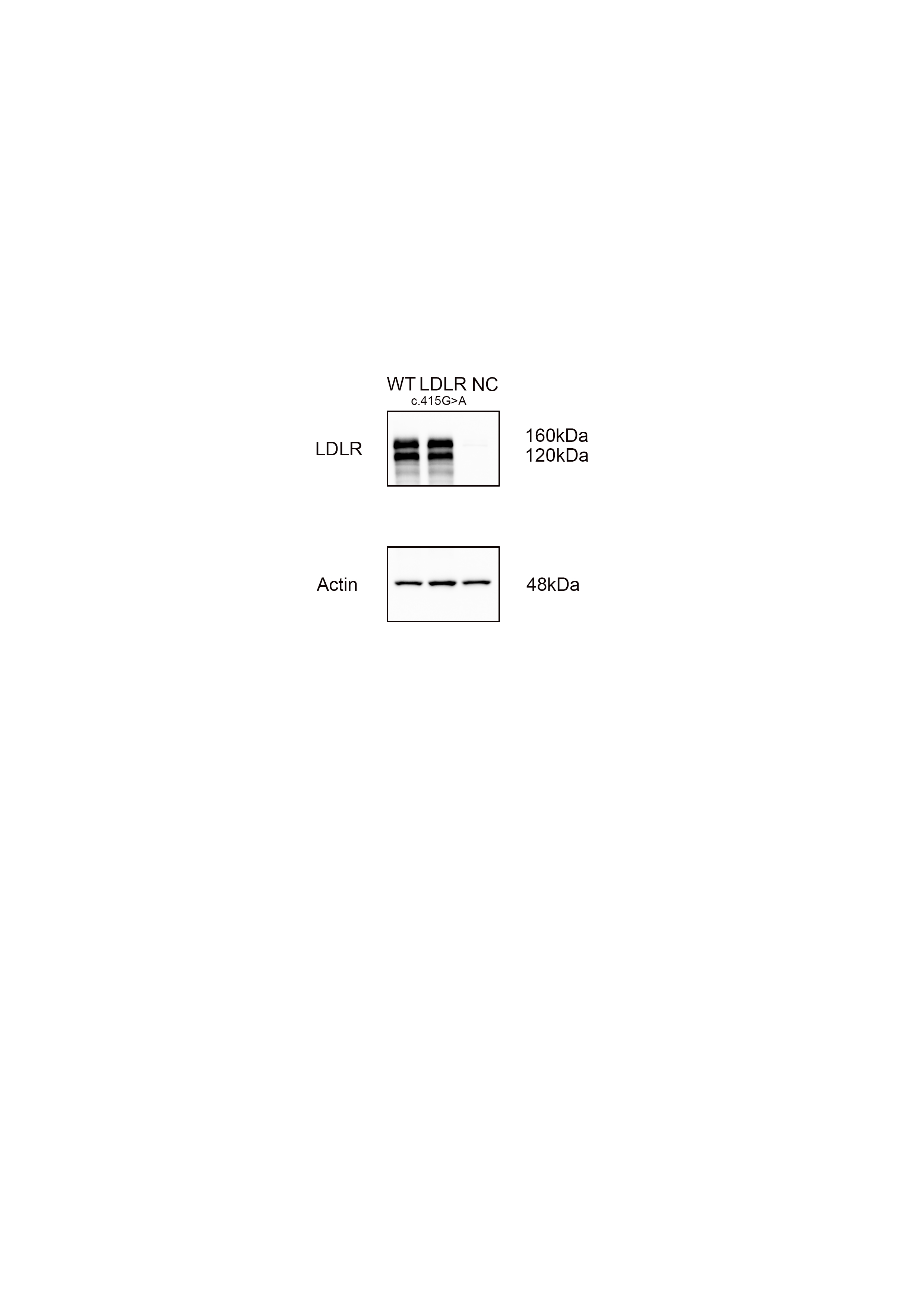

Supplement: Supplementary file 8 — Supplementary Material 8: Western blot analysis of LDLR expression [file 12944_2024_2068_MOESM8_ESM.jpg]
