# Supplementary material for: LDLR c.415G > A causes familial hypercholesterolemia by weakening LDLR binding to LDL
Source: Lipids Health Dis. 2024 Mar 21;23:85. doi: 10.1186/s12944-024-02068-2 (PMC10956282; doi:10.1186/s12944-024-02068-2)
Supplement: Supplementary file 4 — Supplementary Material 4: The proof report of the overall similarity index [file 12944_2024_2068_MOESM4_ESM.pdf]

LDLR c.415G>A causes familial hypercholesterolemia by weakening LDLR binding to LDL

Submission date: 23-Feb-2024 04:08AM (UTC-0500)
Submission ID: 2261384488
File name: Chaessay_report_2664AFC1923B55C3.docx (74.86K)
Word count: 3320
Character count: 18781

LDLR c.415G>A causes familial hypercholesterolemia by weakening LDLR binding to LDL

Kaihan Wang[1#], Tingting Hu[2#], Mengmeng Tai[1#], Yan Shen[1], Haocheng Chai[3], Shaoyi Lin[1*], Xiaomin Chen[1*]

Abstract

Background: Familial hypercholesterolemia (FH) is a prevalent hereditary disease that can cause aberrant cholesterol metabolism. In this study, we confirmed that c.415G>A in low-density lipoprotein receptor (*LDLR*), an FH-related gene, is a pathogenic variant in FH by *in silico* analysis and functional experiments.

Methods: The proband and his family were evaluated using the diagnostic criteria of the Dutch Lipid Clinic Network. Whole-exome and Sanger sequencing were used to explore and validate FH-related variants. *In silico* analyses were used to evaluate the pathogenicity of the candidate variant and its impact on protein stability. Molecular and biochemical methods were performed to examine the effects of the *LDLR* c.415G>A variant *in vitro*.

Results: Four of six participants had a diagnosis of FH. It was estimated that the *LDLR* c.415G>A variant in this family was likely pathogenic. Western blotting and qPCR suggested that *LDLR* c.415G>A does not affect protein expression. Functional studies showed that this variant may lead to dyslipidemia by impairing the binding and absorption of LDLR to low-density lipoprotein (LDL).

Conclusion: *LDLR* c.415G>A is a pathogenic variant in FH; it causes a significant reduction in LDLR's capacity to bind LDL, resulting in impaired LDL uptake. These findings expand the spectrum of variants associated with FH.

Keywords: Familial hypercholesterolemia, Low-density lipoprotein receptor, Pathogenic variant, Functional study

Introduction

Familial hypercholesterolemia (FH) is a hereditary metabolic disease typified by the dysregulation of cholesterol homeostasis [1]. Its principal features are highly elevated plasma low-density lipoprotein cholesterol (LDL-C), xanthoma of the skin and tendon, and the early onset of coronary heart disease [2]. Among patients with FH, a high level of plasma LDL-C is the main driver of cardiovascular risk [3]. In particular, those with homozygous FH may develop atherosclerosis as early as adolescence, affecting not just the arteries but also the valves, resulting in a heavy burden [4]. Clinically, FH is classified as either homozygous or heterozygous; the prevalence of heterozygous FH is about 1:313, while that of homozygous FH is 1:400,000 [5]. However, the early symptoms of FH are easily ignored, making the diagnosis of FH extremely difficult [6]. At present, the diagnosis rate of FH is very low in most countries and regions [7]. For example, it is <10% in the United States [8], 4% in Australia and New Zealand [9], 2% in South Africa [10], and even <1% in Russia, Latin America, and other countries [11]. Only a small percentage of those diagnosed with FH have undergone genetic testing. In most areas, the rate of genetic diagnosis is <5%, such as <5% in the United States [12] and <2% in Asia [13]. Based on the phenomenon of serious complications and the low diagnosis rate of FH, it is urgent to improve the diagnosis rate around the world. Because FH is a genetic disorder, cascade screening based on genetic diagnosis is the most effective way to improve the diagnosis rate [7].

The main pathogenic mechanism underlying FH is the incapacity of LDLR to remove LDL-C from the blood [14]. Under normal physiological conditions, the endoplasmic reticulum produces LDLR, which is then transported to the Golgi apparatus for glycosylation modification and carried to the plasma membrane. Finally, the LDLR on the plasma membrane binds to circulating LDL particles to promote

endocytosis [14]. Once the vesicles encasing the LDLR-LDL complex have been absorbed into the cell, they merge with the endosome. In the acidic endosome, LDLR undergoes a conformational change and separates from the bound LDL [15]. This allows LDLR to return to the plasma membrane for later use or be directed to lysosomes for degradation by interaction with proprotein convertase subtilisin/kexin type 9 (PCSK9) [16]. In addition, LDLR on the cell membrane can also bind to circulating PCSK9 and be carried to lysosomes for degradation [17]. Circulating PCSK9 is secreted from hepatocytes and engages in several biological processes, such as lipid metabolism, immune response, hemostasis, glucose metabolism, and neuronal survival [18]. Among these, the regulation of plasma LDL-C concentrations is the most significant and extensively studied. The activity of PCSK9 is negatively correlated with LDLR density on the surface of hepatocytes and positively correlated with plasma LDL-C concentrations [19]. It has been shown that PCSK9 can form dimers and higher multimers through self-associating, which is influenced by concentration, temperature, and pH, and can increase LDLR degrading activity [20]. Besides, the half-life of circulating PCSK9 might be extended from 5 to 15 minutes by binding to LDLR [21]. Therefore, the increase in PCSK9 expression as well as the increase in activity and the decrease in degradation all lead to a decrease in LDLR, which increases the level of LDL-C in plasma. Any disruption in these processes leads to a notable and significant buildup of LDL-C in the plasm.

Similarly, genetic variants in FH patients cause anomalies in the receptor endocytosis pathway, which abnormally raises plasma levels of LDL-C [22], the extent of which differs between different countries and ethnic groups [23]. Of the previously mentioned genetic variants, variants in the *LDLR* gene account for the bulk of FH instances [24]. During the last few decades, plenty of studies on the *LDLR* variants of

FH have been carried out globally, and many of them have been found in China. For example, in Han Chinese populations, there are 143 different variants of *LDLR* known to exist, and the four most frequent variants include c.986G>A, c.1747C>T, c.1879G>A, and c.268G>A [25, 26]. In Hong Kong, there have been reports of 73 different *LDLR* variants, and the four most common variants included c.1241 T>G, c.1474 G>A, c.769 C>T, and c.1765 G>A [27]. Although more than 4,000 *LDLR* variants have been identified, less than 15% of them have been identified as benign or pathogenic through functional studies [28]. Theoretically, the clinical diagnosis cannot be verified until a genetic variant is identified and subsequently shown to modify the metabolism of LDL [29]. Therefore, it is vital to conduct genetic testing and functional studies on patients with FH, which can provide a strong basis for the diagnosis of FH.

In this study, genetic testing was conducted to identify variants associated with FH in a familial context. Subsequent *in silico* analysis and *in vitro* functional assessments were performed to identify the pathogenicity of *LDLR* c.415G>A. These findings contribute to broadening the spectrum of FH-related variants, thereby facilitating early diagnosis.

Methods

Study participants and blood sample collection

According to the diagnostic criteria of the Dutch Lipid Clinic Network (DLCN), individuals with scores of ≥ 8 points and their families were included in this study. After using DLCN diagnostic criteria to evaluate the participants, a family tree was built. Venous blood was collected from participants for a blood lipid analysis and subsequent

whole-exome sequencing. Participants had completed informed consent forms, which were authorized by the First Affiliated Hospital Ethics Committee of Ningbo University.

Whole-exome sequencing

Venous blood samples were forwarded to the Beijing Genomics Institution (BGI, Wuhan, China) for whole-exome sequencing. After low-quality reads, adapters, and a high percentage of N-bases were removed from the raw sequencing data, alignments against the human reference genome hg19 sequence were generated using Burrows Wheeler Aligner [30]. Using the Genome Analysis Toolkit (GATK), duplicate reads were tagged, and base mass value recalibration was performed. GATK4's HaplotypeCaller was used to find single nucleotide polymorphisms (SNPs) and InDels [31]. Rigorous filtering was applied to extract SNPs and InDels that are both highly dependable and of excellent quality.

Sanger sequencing

Utilizing the E Z. N.P. ® Blood DNA Mini Kit (D3392-02; Omega Bio-Tek, Norcross, GA, USA) for DNA extraction, which was then amplified using polymerase chain reaction (PCR). The total PCR system was 50 μL, which includes 25 μL of 2× ES Taq Master Mix, 2 μL of forward primer (5′-CAGGACGAGTTTCGCTGCCAC-3′), 2 μL of reverse primer (5′-ATCCGAGCCATCTTCGCAGTC-3′), 500 ng of DNA and enzyme-free water. After sending the PCR products to BGI for Sanger sequencing, data analysis was conducted using Chromas software.

***In silico* analysis**

MutationTaster was used to predict the pathogenicity of point variants [32]. DynaMut was utilized to evaluate how point variants affected the stability and flexibility of proteins [33]. A normal mode analysis was used to determine the

difference in free energy change (ΔΔG) between the structures of the wild-type (WT) and the variant. ENCoM-based difference in vibrational entropy (ΔΔSVib) was used to predict the difference in flexibility [34]. SnapGene v6.0.2 was employed to determine the conservation of protein sequences among species using the multiple sequence comparison by log-expectation (MUSCLE) algorithm.

Plasmid construction, cell culture, and transfection

Shanghai GeneChem Co. (Shanghai, China) completed the construction of human WT *LDLR* and *LDLR* c.415G>A with a FLAG epitope close to the N terminus in the GV208 vector. HEK293T cells were used for the plasmid transfection [35]. The cells were grown in Eagle media that had been modified by Dulbecco (high glucose) (Cytiva, Shanghai, China) containing 10% fetal bovine serum (Vivacell, Shanghai, China). For transfection, the cells were transfected with 2500 ng of plasmid DNA using Lipofectamine™ 3000 Reagent (Invitrogen, Shanghai, China) in a six-well plate.

Quantitative real-time PCR

Following transfection, TRIzol (Omega, Norwalk, CT, USA) was used to extract RNA, and the HiFiScript cDNA Synthesis Kit (CW2569M; CWBIO, Beijing, China) was used for reverse transcription. The Mastercycler® Nexus X2 (Eppendorf, Hamburg, Germany) was used to carry out quantitative real-time PCR (qPCR). TaqMan assays were employed for the detection of fluorescence. The relative amplification efficiency of *LDLR* was established using the comparative Ct method. The primers used were as follows: *LDLR*, F-5′-AAGTGCATCTCTCGGCAGTT-3′, *LDLR*, R-5′-CCACTCATCCGAGCCATCTT-3′; *GAPDH*, F-5′-GGAAATCGTGCGTGACATTA-3′, R-5′-GGAAGGAAGGCTGGAAGAG-3′.

Western blotting

The cells were lysed using RIPA solution (Solarbio, Beijing, China), which contains inhibitors of phosphatase and protease. The proteins were boiled for 10 min with loading buffer (Solarbio, Beijing, China) in preparation for western blotting. Following 7.5% SDS/PAGE resolution, the samples were blotted onto PVDF membranes (Merck, Darmstadt, Germany). After using 5% skim milk to prevent non-specific binding, monoclonal mouse anti-FLAG (1:3000, F1804; Sigma, Shanghai, China) and monoclonal rabbit anti-β-actin (1:10000, AF7018; Affinity Biosciences, San Francisco, California, USA) primary antibodies were added, and the mixture was incubated for a whole night at 4°C. Then, the samples were treated with the corresponding horseradish peroxidase-conjugated IgG for 60 min. Lastly, the immunoreactive proteins were identified using enhanced chemiluminescence.

Flow cytometry

Cells were added to a six-well plate with 0.05% trypsin and transferred into a 2 mL EP tube. Diluted rabbit anti-human LDLR monoclonal antibody conjugated with allophycocyanin (1:200, ab275614; Abcam, Cambridge, MA, USA) was added, and the mixture was maintained in the dark for an additional hour after blocking with 10% donkey serum for an hour at room temperature. The mean fluorescence levels from at least three replicate estimates were obtained using a Beckman CytoFlex S flow cytometer (Beckman Coulter, Shanghai, China). Data analysis was done with FlowJo software.

Immunofluorescence

After transfection, cells were fixed using 4% paraformaldehyde (P1110; Solarbio,

Beijing, China). Following a wash with 1× PBS, the cells were blocked using a 10% goat serum solution to prevent non-specific binding. Next, mouse anti-flag antibody (1;3000, F1840; Sigma-Aldrich, Saint Louis, USA) was diluted in 1x PBS and incubated at 4°C for 4 h, along with 20 µg/mL labelled human plasma LDL (Dil-LDL; L3482; Thermo Fisher, Shanghai, China). After incubation, the cells were washed with 1× PBS and then treated with goat anti-mouse IgG conjugated with AlexaFluor488 (1:500, ab150113; Abcam, Cambridge, UK). After completing the staining of nucleus with 4′,6-diamidino-2-phenylindole (DAPI), the cells can be observed under a LEICA TCS SP8 confocal laser scanning microscope.

In order to assess the uptake capacity of LDLR, transfected HEK293T cells were treated with 20 µg/mL Dil-LDL for 4 h at 37°C. Similarly, confocal microscopy was used for analysis after washing with PBS, fixation with 4% paraformaldehyde, and DAPI labeling of cell nuclei.

Statistical analysis

All data was analyzed using GraphPad Prism (version 9.0.0; La Jolla, CA), and presented as means ± SEM. Normal distribution was evaluated using the D'Agostino–Pearson omnibus normality test. Group differences were evaluated using a one-way ANOVA. $P < 0.05$ was used as the statistical significance criterion.

Results

Clinical data for the proband and his family members

The proband, a 39-year-old male who presented to the First Affiliated Hospital of Ningbo University due to chest tightness following physical activity. Coronary angiography revealed that his coronary artery was severely stenotic. Because of the early onset of atherosclerotic cardiovascular disease, FH was suspected; therefore,

cascade screening was conducted. The biochemical results and DLCN scores for this family member are shown in Tables 1 and 2. Figure 1 depicts the pedigree. A positive family history of dyslipidemia was found in the pedigree analysis of the index case, which is compatible with an autosomal dominant mode of inheritance.

Genetic analysis and *in silico* screening

Owing to geographical constraints, blood samples were only obtained from three family members for whole-exome sequencing. The whole-exome sequencing data (Supplemental table 1) were analyzed for variants in FH-related genes (*LDLR*, *APOB*, *PCSK9*, *LDLRAP1*). Two patients with FH in this family were identified to carry the missense variant *LDLR* c.415G>A (Figure 2A). The presence of *LDLR* c.415G>A, which is found in exon 4 of the *LDLR* gene on chromosome 19 p13.2, was verified using Sanger sequencing (Figure 2B). An interspecific sequence analysis revealed that the altered amino acid sequence is highly conserved (Figure 2C). A MutationTaster analysis showed that the variant is pathogenic.

Additionally, the interatomic interactions of *LDLR* c.415G>A were assessed using DynaMut. The differences in interatomic interactions between the WT and variant are depicted in Figure 2D. According to the predicted DynaMut ΔΔG values and ΔΔS ENCoM (Empirical Normal-Coordinate Analysis Method), the variant resulted in decreased molecular flexibility and increased stability of the LDLR protein.

LDLR* c.415G>A variant does not change LDLR expression *in vitro

To confirm LDLR c.415G>A's effect on gene expression, HEK293T cells were transfected with plasmids carrying WT *LDLR*, variant *LDLR*, and blank. According to the immunofluorescence results, the transfection success rate was approximately 85% (Supplemental Figure 1). qPCR results, as illustrated in Figure 3A, demonstrated that

cells transfected with variant plasmids did not exhibit any differences in *LDLR* mRNA expression compared with those transfected with WT plasmids, whereas cells transfected with blank plasmids exhibited extremely low expression of *LDLR* mRNA. Western blotting results (Figure 3B) revealed that LDLR protein expression was similar in the variant and WT groups but was essentially absent in the blank group. The flow cytometry results, as shown in Figure 3C, demonstrated that the cell membrane in the blank group did not express the LDLR protein, while cell membranes in the variant and WT groups exhibited similar LDLR protein expression levels. The expression levels of the variant and WT groups did not differ statistically in any appreciable way. These findings show that gene expression is unaffected by the *LDLR* c.415G>A variant.

***LDLR* c.415G>A decreases Dil-LDL absorption by cells**

To examine if *LDLR* c.415G>A impacts protein activity, plasmid-transfected cells were co-incubated with Dil-LDL at 37°C for at least 4 h. The variant group's red fluorescence was much less than that of the WT group, as shown in Figure 4, suggesting that the variant group's LDL uptake was noticeably lower than that of the WT group. The capacity to absorb LDL in the blank control group was minimal. These results indicate that *LDLR* c.415G>A impaired the capacity to absorb LDL significantly.

***LDLR* c.415G>A weakens the ability of LDLR to bind to LDL**

The mechanism underlying the lower absorption ability induced by the *LDLR* c.415G>A variant was further evaluated using laser confocal microscopy to investigate the ability of LDLR to bind to LDL after co-incubating the plasmid-transfected cells with LDL antibodies and Dil-LDL at 4°C for 4 h. As shown in Figure 5, although there was a significant decrease in LDL binding, the LDLR protein content in the variant group was nearly equal to that of the WT group. Therefore, by decreasing LDLR

binding to LDL, the variant dramatically lowers the absorption capacity.

Discussion

In this study, serious issues in lipid metabolism were observed in the proband and his son, who carried *LDLR* c.415G>A. This variant was previously described [36] and was included in the ClinVar database. (NM_000527.4(LDLR):c.415G>A (p.Asp139Asn)) as likely pathogenic with accession number RCV000237450.1, but no functional studies have been conducted. The present functional studies revealed that while this variant had no effect on protein synthesis, it dramatically lowered LDL absorption via impairing the ability of LDLR to bind LDL. It is hypothesized that this variant affects the uptake ability of LDLR, inhibiting the regular excretion of LDL-C in plasma and resulting in FH.

Mature LDLR proteins consist of 860 amino acids and can be divided into five functional domains [37]. Among these, the interaction between acidic residues of the LDLR ligand-binding domain and basic residues of apoB100 mediates the binding of LDL to LDLR [38]. The variant detected in this study is located in the ligand-binding domain. The replacement of asparagine with aspartic acid results in distinct molecular interactions with the surrounding residues. This may explain the lack of affinity of the variant protein for LDL. Previous functional studies of pathogenic of *LDLR* variants have revealed that *LDLR* p.L799R disrupts the transmembrane domain, inhibiting membrane insertion and resulting in the secretion of LDLR [39]. *LDLR* p. D482H and C667F were trapped in the endoplasmic reticulum owing to misfolding [40]. *LDLR* p.W23X, S78X, or W541X nonsense mutations significantly decreased the levels of mRNA expression [41]. To sum up, *LDLR* variants can lead to FH by altering different

stages of receptor-mediated endocytosis. Certain circumstances may result in the total absence of receptors, whereas other circumstances may result in receptors that are present but with impaired function. All these will lead to the inability of cells to absorb LDL, which will build up cholesterol in the blood and raise the risk of atherosclerosis. In this family, both the proband and his son exhibited serious problems related to lipid metabolism, although genetic testing showed that they were heterozygous and cell tests demonstrated that the variant did not affect protein expression. Lipid metabolism is closely related to a decline in receptor function.

Even though FH is the most common disease associated with disorders in cholesterol metabolism, it has received fairly little public attention, and its rate of diagnosis is quite poor [6]. Most patients with FH do not receive an effective lipid-lowering medication [42]. Since early myocardial infarction, stroke, and an elevated risk of overall mortality are frequent features of untreated FH, it is well acknowledged that the illness poses a serious risk to life [43]. Therefore, more research is required to improve outcomes for patients with FH and their families.

Study strengths and limitations

The pathogenicity of missense variant *LDLR* c.415G>A was confirmed by this study, which impaired binding and uptake of LDLR to LDL. These findings underpin the early diagnosis of FH, contribute to cascade screening of FH families, and advocate for personalized treatment strategies. However, this study still had some limitations. Firstly, no *in vivo* functional experiments were conducted. Further evidence from gene-edited murine models to confirm the pathogenicity of the *LDLR* c.415G>A variant is required. Secondly, the assessment of LDLR activity relied solely on one cell line model system,

necessitating validation across diverse cell line models to ensure research robustness. Finally, the scope of the current study did not extend to investigating how to address the pathogenicity of this variant. Future studies are required to ameliorate the harmful consequences of variants, which will help achieve more effective lipid-lowering treatments.

Conclusion

LDLR c.415G>A is a pathogenic variant in FH. It causes acidic amino acids to be replaced, greatly reducing the capacity of LDLR to bind to LDL. This prevents LDL-C from being taken up by cells and produces a noticeable LDL-C increase in plasma. This study advances our understanding of FH-associated gene variants and identifies a pathogenic variant, providing information that contributes to the study of early diagnosis and treatment of FH.

LDLR c.415G>A causes familial hypercholesterolemia by weakening LDLR binding to LDL

ORIGINALITY REPORT

9%	5%	5%	2%
SIMILARITY INDEX	INTERNET SOURCES	PUBLICATIONS	STUDENT PAPERS

PRIMARY SOURCES

1	www.ncbi.nlm.nih.gov<br>Internet Source	2%
2	Shaoyi Lin, Tingting Hu, Kaihan Wang, Jiaqi Wang, Yunyun Zhu, Xiaomin Chen. "In vitro assessment of the pathogenicity of the LDLR c.2160delC variant in familial hypercholesterolemia", Lipids in Health and Disease, 2023<br>Publication	2%
3	Ya-nan Hu, Min Wu, Yuan-yuan Ji, Jian-Hui Zhang et al. "Analysis of Low-Density Lipoprotein Receptor Gene Mutations in A Family with Familial Hypercholesterolemia", Research Square Platform LLC, 2022<br>Publication	1%
4	www.nature.com<br>Internet Source	1%
5	Asier Benito-Vicente, Kepa Uribe, Shifa Jebari, Unai Galicia-Garcia, Helena Ostolaza, Cesar Martin. "Validation of LDLr Activity as a Tool	<1%

to Improve Genetic Diagnosis of Familial Hypercholesterolemia: A Retrospective on Functional Characterization of LDLr Variants", International Journal of Molecular Sciences, 2018
Publication

6	Submitted to University of Lincoln<br>Student Paper	<1 %
7	Submitted to University of South Florida<br>Student Paper	<1 %
8	www.spandidos-publications.com<br>Internet Source	<1 %
9	docksci.com<br>Internet Source	<1 %
10	www.mdpi.com<br>Internet Source	<1 %
11	jasbsci.biomedcentral.com<br>Internet Source	<1 %
12	pubmed.ncbi.nlm.nih.gov<br>Internet Source	<1 %
13	Submitted to HTM (Haridus- ja Teadusministeerium)<br>Student Paper	<1 %
14	mgend.med.kyoto-u.ac.jp<br>Internet Source	<1 %

15	www.omicsdi.org<br>Internet Source	<1 %
16	www.researchsquare.com<br>Internet Source	<1 %
17	www.unboundmedicine.com<br>Internet Source	<1 %

Exclude quotes	Off	Exclude matches	Off
Exclude bibliography	On		
